# The Association Between Estimated Glomerular Filtration Rate and Hospitalization for Fatigue: A Population-Based Cohort Study

**DOI:** 10.1177/20543581211001224

**Published:** 2021-03-16

**Authors:** Janine F. Farragher, Jianguo Zhang, Tyrone G. Harrison, Pietro Ravani, Meghan J. Elliott, Brenda Hemmelgarn

**Affiliations:** 1 Department of Community Health Sciences, University of Calgary, AB, Canada; 2 Department of Medicine, University of Calgary, AB, Canada; 3 O’Brien Institute for Public Health, Cumming School of Medicine, University of Calgary, AB, Canada; 4 Faculty of Medicine & Dentistry, University of Alberta, AB, Canada

**Keywords:** chronic kidney disease, dialysis, fatigue, hospitalization

## Abstract

**Background:**

Fatigue is a pervasive symptom among patients with chronic kidney disease (CKD) that is associated with several adverse outcomes, but the incidence of hospitalization for fatigue is unknown.

**Objective:**

To explore the association between estimated glomerular filtration rate (eGFR) and incidence of hospitalization for fatigue.

**Design:**

Population-based retrospective cohort study using a provincial administrative dataset.

**Setting:**

Alberta, Canada.

**Patients:**

People above age 18 who had at least 1 outpatient serum creatinine measurement taken in Alberta between January 1, 2009, and December 31, 2016.

**Measurements:**

The first outpatient serum creatinine was used to estimate GFR. Hospitalization for fatigue was identified using International Classification of Diseases, Tenth Revision (ICD-10) code R53.x.

**Methods:**

Patients were stratified by CKD category based on their index eGFR. We used negative binomial regression to determine if there was an increased incidence of hospitalization for fatigue by declining kidney function (reference eGFR ≥ 60 mL/min/1.73m^2^). Estimates were stratified by age, and adjusted for age, sex, socioeconomic status, and comorbidity.

**Results:**

The study cohort consisted of 2 823 270 adults, with a mean age of 46.1 years and median follow-up duration of 6.0 years; 5 422 hospitalizations for fatigue occurred over 14 703 914 person-years of follow-up. Adjusted rates of hospitalization for fatigue increased with decreasing kidney function, across all age strata. The highest rates were seen in adults on dialysis (adjusted incident rate ratios 24.47, 6.66, and 3.13 for those aged 18 to 64, 65 to 74, and 75+, respectively, compared with eGFR ≥ 60 mL/min/1.73m^2^).

**Limitations:**

Fatigue hospitalization codes have not been validated; reference group limited to adults with at least 1 outpatient serum creatinine measurement; remaining potential for residual confounding.

**Conclusions:**

Declining kidney function was associated with increased incidence of hospitalization for fatigue. Further research into ways to address fatigue in the CKD population is warranted.

**Trial Registration:**

Not applicable (not a clinical trial).

## Abrégé

**Contexte:**

La fatigue est un symptôme généralisé et associé à plusieurs effets indésirables chez les patients atteints d’insuffisance rénale chronique (IRC). L’incidence des hospitalisations liées à la fatigue est néanmoins inconnue.

**Objectif:**

Examiner l’association entre le débit de filtration glomérulaire estimé (DFGe) et l’incidence d’hospitalization pour fatigue.

**Type d’étude:**

Étude de cohorte rétrospective représentative d’une population et menée avec un ensemble de données administratives provinciales.

**Sujets:**

Les patients adultes de l’Alberta ayant eu au moins une mesure de créatinine sérique en consultation externe entre le 1er janvier 2009 et le 31 décembre 2016.

**Mesures:**

La première mesure du taux de créatinine sérique en consultation externe a été utilisée pour estimer le DFG. L’hospitalization pour fatigue a été définie avec le code CIM-10 R53.x.

**Méthodologie:**

Les patients ont été stratifiés par catégorie d’IRC en fonction de l’indice de DFGe. Une régression binomiale négative a servi à déterminer si le déclin de la fonction rénale (référence: DFGe ≥ 60 mL/min/1,73 m ^2^) menait à une incidence accrue d’hospitalization pour fatigue. Les estimations ont été stratifiées selon l’âge et corrigées en fonction de l’âge, du sexe, du statut socio-économique et des comorbidités.

**Résultats:**

L’étude porte sur 2 823 270 adultes (âge moyen: 46,1 ans) dont la durée médiane de suivi s’établissait à 6,0 ans. Au cours des 14 703 914 années-personnes de suivi, 5 422 hospitalisations pour fatigue ont été répertoriées. Les taux corrigés d’hospitalization pour fatigue ont augmenté avec le déclin de la fonction rénale pour toutes les strates d’âges. Les taux les plus élevés ont été observés chez les patients dialysés (rapports des taux d’incidents corrigés: 24,47 [patients de 18 à 64 ans]; 6,66 [patients de 65 à 74 ans] et 3,13 [patients de 75 ans et plus] comparativement à un DFGe ≥ 60 mL/min/1,73 m^2^).

**Limites:**

Les codes d’hospitalization pour fatigue n’ont pas été validés; le groupe de référence a été limité aux adultes ayant au moins une mesure de créatinine sérique en consultation externe; possibilité de facteurs de confusion résiduels.

**Conclusion:**

Le déclin de la fonction rénale a été associé à une incidence accrue d’hospitalization pour fatigue. Ces résultats justifient de poursuivre les recherches sur les moyens de remédier à la fatigue chez les patients atteints d’IRC.

## Introduction

Chronic kidney disease (CKD) is increasingly recognized as a global health problem that affects approximately 1 in 9 adults worldwide.^1^ Fatigue is among the most common symptoms of CKD, experienced by 49% to 100% of the non-dialysis CKD population^2^ and about 2 in 3 people with end-stage kidney disease (ESKD) receiving dialysis.^3^ Fatigue describes an extreme tiredness that is disproportionate to activity or exertion, and interferes with daily living.^4^ The pervasive impact of fatigue on the lives of people with CKD and ESKD has become evident in several recent studies. It has been described by patients on dialysis as a “debilitating and exhausting burden” that leads to restricted life participation,^5,6^ and has been identified as a top priority for CKD and ESKD research in priority-setting exercises with patients.^7,8^ Fatigue is associated with several factors in CKD, such as anemia, inflammation, mood disorders, physical inactivity, poor sleep, and dialysis-related factors. It has been shown to be associated with several poor outcomes in this population, including reduced independence in daily activities^5,6,9,10^ and increased mortality^11^ among people with ESKD, and reduced quality of life among people with CKD.^12^ However, the association of being hospitalized for fatigue in people with CKD and ESKD is an underexplored research area.

There are several reasons why people with CKD might be at a greater risk of being hospitalized for fatigue compared with those without CKD. First, people with CKD are known to experience hospitalizations more frequently than the general population. Estimates suggest that people with category 3a CKD have a 10% increased rate of hospitalization compared with the general population, while people with categories 3b, 4, and 5 CKD experience 50%, 110%, and 210% increased rates of hospitalization, respectively.^13^ Hospitalizations are especially frequent in the ESKD population on dialysis, occurring an estimated 1.7 times per patient-year. Furthermore, a recent study in Finland found that 4.5% of all emergency department visits among octogenarians were related to nonspecific malaise and fatigue.^14^ Although hospitalizations for fatigue have not been reported in chronic disease populations to our knowledge, the fact that the CKD population is predominantly geriatric suggests this may be an important issue for this population. The objective of this study was therefore to explore the association between estimated glomerular filtration rate (eGFR) and hospitalizations for fatigue, using a population-based cohort from Alberta, Canada.

## Materials and Methods

### Study Design and Population

We conducted a population-based cohort study, using the Alberta Kidney Disease Network (AKDN) repository of laboratory and administrative data from Alberta, Canada. The AKDN data include laboratory test results; socio-demographic data; and clinical data including comorbidities, health care encounters, death, and kidney-related outcomes, on all adults from the province of Alberta.^15^ The study cohort was comprised of all adults aged 18 or older, residing in Alberta, with at least 1 outpatient serum creatinine measurement taken between January 1, 2009, and December 31, 2016. The study index date was the date of each participant’s first outpatient serum creatinine measurement, date of dialysis initiation, or January 1, 2009, for those already on dialysis as of January 1, 2009.

### Measurement of Kidney Function

Serum creatinine measurements for the study cohort were obtained from provincial laboratories in Alberta, where measurement practices are standardized across laboratories. The index outpatient serum creatinine measurement was used to estimate kidney function, and eGFR was calculated according to the 4-variable CKD-EPI study equation.^16^ Patients were stratified by category of kidney function: eGFR ≥60 mL/min/1.73m^2^ (reference); 45-59 mL/min/1.73m^2^; 30-44 mL/min/1.73m^2^; 15-29 mL/min/1.73m^2^; <15 mL/min/1.73m^2^ (no dialysis); or dialysis (hemodialysis or peritoneal dialysis).

### Covariates

Covariates included age, sex, socioeconomic status, and Charlson comorbidities (myocardial infarction, congestive heart failure, peripheral vascular disease, cerebrovascular disease, dementia, chronic pulmonary disease, connective tissue disorder, peptic ulcer disease, mild liver disease, diabetes, diabetes with chronic complication, hemiplegia/paraplegia, any malignancy without metastases, leukemia, lymphoma, moderate or severe liver disease, metastatic solid tumor, and HIV). Age and sex were derived from the administrative database of the Alberta Health registry file. Socioeconomic status was ascertained using the most recent Canadian Census data to determine median household income in the patient’s geographical area, and patients in the bottom income quintile were categorized as having a low socioeconomic status. Charlson comorbidities were defined using validated algorithms from hospital discharge records and physician claims. The presence of 1 or more diagnostic code in any position up to 3 years prior to cohort entry were used to identify the comorbidities, except for diabetes and hypertension, where other validated algorithms were used.^17,18^

### Outcome Variable

The primary outcome was the rate of fatigue-related hospitalizations. Fatigue-related hospitalizations were defined as all hospitalizations assigned the following fatigue-related International Classification of Diseases, Tenth Revision (ICD-10) codes as the most responsible discharge diagnosis from the Alberta Health Inpatient Encounters database: R53.0 (neoplastic [malignant] related fatigue), R53.1 (weakness), or R53.8.x (other malaise and fatigue). In Canada, ICD-10 codes are assigned by health information management specialists (“coders”) via a review of information documented by physicians in patients’ health records. Coders follow coding standards developed by the Canadian Institutes of Health Information. Hospitalizations coded with R53.2 (functional quadriplegia) were excluded, due to their irrelevance to CKD. We also conducted a sensitivity analysis to explore rates of hospitalizations in the CKD population where fatigue was coded as a primary or secondary discharge diagnosis.

### Statistical Analysis

Baseline characteristics of the study cohort, by eGFR categories, were described using frequencies (percentages) and/or means (standard deviations), where appropriate. We hypothesized that lower levels of eGFR would be associated with higher rates of hospitalization for fatigue. To test this, we used negative binomial regression to estimate the rates of fatigue-related hospitalizations per 1000 person-years, by eGFR category. We also used negative binomial regression to assess the association between CKD category (with eGFR > 60 mL/min/1.73m^2^ as the reference) and hospitalizations for fatigue. Because our hypothesis that age would modify the association between eGFR category and hospitalizations for fatigue was confirmed (*P* < .001 for interaction), we reported the rate of fatigue-related hospitalizations for each eGFR category stratified by age group (<65, 65-74, and ≥75 years). All analyses were adjusted for age, sex, socioeconomic status, diabetes, hypertension, and the Charlson comorbidities. Patients were followed from their index date to the earliest of the study end date (December 31, 2016), date of kidney transplantation, death, or out-migration from the province. Characteristics of fatigue-related hospitalizations, including secondary discharge diagnoses and discharge disposition of hospitalized patients, were also described.

Statistical analyses were conducted using SAS version 9.4 and STATA version 14.0.

## Results

We identified 2 823 270 people who had at least 1 outpatient serum creatinine measurement or were established on maintenance dialysis during the study period, and who formed the study cohort. Participants were followed for a median duration of 6.0 years (interquartile range [IQR] = 3.3-7.3).

Baseline characteristics of the cohort are described in Table 1. The mean age of the cohort was 46.1 years, and 54% were female. A total of 150 894 participants (5.3%) had a baseline eGFR below 60 mL/min/1.73m^2^. Those with eGFR <60 mL/min/1.73m^2^ were predominantly older, male, and more likely to experience comorbidities including diabetes, cardiovascular disease, and cancer compared with the reference group.

**Table 1.** Baseline Characteristics of Study Population by Category of eGFR (in mL/min/1.73m^2^).

Variable	Total (N = 2 823 270)	CKD 1 & 2: eGFR ≥60 (n = 2 672 376)	CKD 3a: 45 ≤ eGFR < 60 (n = 100 550)	CKD 3b: 30 ≤ eGFR < 45 (n = 36 123)	CKD 4: 15 ≤ eGFR < 30 (n = 10 579)	CKD 5: eGFR < 15, no dialysis (n = 1812)	CKD 5: Dialysis (n = 1830)
Age (mean [SD])	46.1 (17.1)	44.5 (15.9)	72.2 (12.9)	77.2 (12.6)	76.3 (14.8)	67.8 (18.1)	62.6 (16.6)
Female	53.9	53.8	55.9	58.5	56.5	46.2	39.7
Rural residence	11.1	11.0	13.4	13.0	12.8	12.1	12.0
Income <20th percentile	23.2	23.0	25.9	27.8	29.1	27.4	30.0
Hypertension	22.1	19.2	69.1	84.3	87.1	77.9	88.7
Diabetes	7.5	6.5	21.0	30.3	38.9	38.9	51.3
Cancer	4.2	3.7	10.7	13.0	14.5	14.1	16.5
Cerebrovascular Disease	1.8	1.4	7.5	11.3	12.9	12.0	14.0
Congestive heart failure	1.7	1.1	9.6	19.6	28.4	25.2	33.1
Chronic obstructive pulmonary disease	11.3	10.9	18.2	22.8	25.3	20.2	26.0
Dementia	1.2	0.7	6.8	11.6	13.3	10.5	7.0
HIV/AIDS	0.1	0.1	0.1	0.1	0.0	0.1	0.1
Metastatic solid tumor	0.5	0.5	1.3	1.9	2.4	2.4	3.2
Myocardial infarction	1.6	1.2	6.4	10.4	13.7	13.2	20.7
Mild liver disease	0.8	0.8	1.1	1.4	1.8	2.4	3.6
Moderate/severe liver disease	0.1	0.1	0.2	0.4	0.7	1.2	1.7
Hemiplegia or paraplegia	0.3	0.3	0.7	1.2	1.4	1.3	2.2
Peptic ulcer disease	1.3	1.2	2.5	3.4	4.2	4.4	8.0
Peripheral vascular disease	1.0	0.7	4.8	8.0	11.1	8.4	35.2
Rheumatologic disease	1.1	1.0	2.6	3.4	4.0	2.7	5.8

*Note.* All data expressed as % unless otherwise noted. eGFR = estimated glomerular filtration rate; CKD = chronic kidney disease; SD = standard deviation.

**Figure 1.**
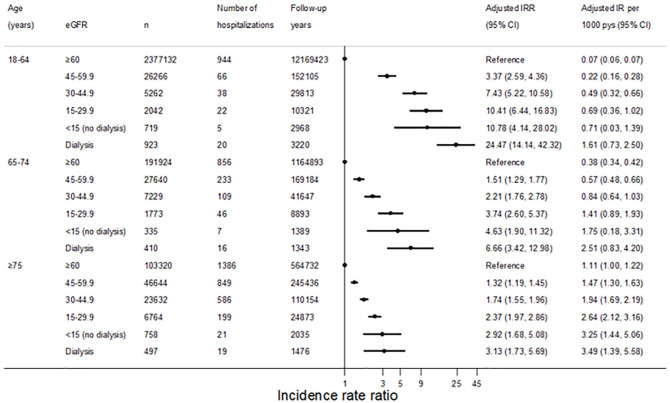
Incidence of hospitalization for fatigue, stratified by age and kidney function. *Note.* eGFR = estimated glomerular filtration rate; IRR = incidence rate ratio; CI = confidence interval; IR = incidence rate.

There were a total of 5422 hospitalizations for fatigue among 2 823 270 patients, that occurred over 14 703 914 person-years of follow-up (crude incidence rate [IR] 0.37 per 1000 person-years, 95% confidence interval [CI], 0.36-0.38). Hospitalizations for fatigue occurred most frequently in people aged ≥75 years (crude IR 3.23 per 1000 person-years [95% CI, 3.11-3.34]), and least frequently in those aged 18 to 64 (crude IR 0.089 per 1000 person-years [95% CI, 0.083-0.094]). Across all age groups, the adjusted rate of hospitalizations for fatigue increased with decreasing kidney function (Figure 1). Compared with the reference group, the association between eGFR and hospitalization for fatigue was most pronounced in those aged 18 to 64 years, and attenuated in the older age groups. In those aged 18 to 64 years, the adjusted incidence rate ratio (IRR) of hospitalizations for fatigue compared with the reference population was 3.37 (95% CI, 2.59-4.36) in those with a baseline eGFR of 45 to 60 mL/min/1.73m^2^, and 7.43 (95% CI, 5.22-10.58) in those with a baseline eGFR of 30 to 45 mL/min/1.73m^2^. By comparison, the adjusted IRR in those aged ≥75 years was 1.32 (95% CI, 1.19-1.45) in those with eGFR 45 to 60 mL/min/1.73m^2^, and 1.74 (95% CI, 1.55-1.96) in those with eGFR 30 to 45 mL/min/1.73m^2^. People on maintenance dialysis had the highest risk of hospitalization for fatigue across the 3 age categories: dialysis was associated with a 3.1-fold increase in hospitalizations for fatigue in people above the age of 75 years; a 6.7-fold increase in those aged 65 to 74 years; and a 24-fold increase in adults aged 18 to 64 years. Crude IRs of hospitalizations with fatigue coded as a primary or secondary discharge diagnosis are provided in Supplement 1.

Of 2065 people with CKD who were hospitalized for fatigue, 271 (13%) experienced more than 1 such hospitalization. The median length of stay of hospitalizations for fatigue in people with CKD was 8 days (IQR 3-20), while the most common secondary discharge diagnoses used were repeated falls (7.7%), dehydration (5.2%), and urinary tract infection (5.1%); 37% of people hospitalized for fatigue with CKD were discharged home without support services, while 33% were discharged home with support services, 16% were transferred to continuing care, and 6% died in hospital (Table 2).

**Table 2.** Discharge Disposition After Hospitalization for Fatigue in Adults With Estimated Glomerular Filtration Rate <60 mL/min/1.73m^2^.

Discharge disposition	Frequency (n = 2065)	%
Transferred to acute care inpatient institution	110	5.33
Transferred to continuing care	338	16.37
Transferred to other	31	1.50
Discharged home with support services	689	33.37
Discharged home	755	36.56
Sign-out	19	0.92
Death	121	5.86
Did not return from pass	2	0.10

## Discussion

Using a large population-based cohort, we found that reduced eGFR was associated with an increased risk of hospitalization for fatigue. Rates of hospitalization for fatigue increased with decreasing kidney function across all age strata, and were most pronounced compared with the reference population in the youngest age subgroups. Even people with the least severe degree of CKD (eGFR 45-60 mL/min/1.73m^2^) experienced statistically significant increased incidence of hospitalization for fatigue, ranging from 1.3- to 3.4-fold higher than those with normal kidney function (ie, eGFR > 60 mL/min/1.73m^2^). People on dialysis displayed the greatest incidence of hospitalization for fatigue, ranging from 3.1- to 24.5-fold higher than the reference population when adjusted for important clinical and demographic variables.

Previous studies exploring fatigue and health outcomes in patients with CKD described fatigue as a common symptom of ESKD that interferes with day-to-day functioning.^3,5,6^ Survey data also showed fatigue to be a common symptom in higher categories of eGFR,^19-21^ although little else had been reported about the impact of fatigue in earlier stages of CKD. Our study provides additional perspective on the burden of fatigue in CKD and ESKD, suggesting that at times fatigue may result in hospitalization, although the frequency of these events was low across ages and categories of CKD. Despite the relatively low rates of hospitalizations for fatigue, we nonetheless argue that any hospitalization event is significant, as they are associated with an increased risk of subsequent functional decline^22^ and other poor outcomes, especially in the elderly. Furthermore, we speculate whether a proportion of these hospitalizations could be prevented with timely and proactive intervention for fatigue in the CKD and ESKD populations.

Among the factors that could contribute to hospitalizations for fatigue in CKD is an under-recognition of the burden of patient fatigue in routine CKD care. Patient-reported outcomes such as functional status, quality of life, and fatigue are typically not assessed regularly in people with CKD or ESKD.^6^ Without timely assessment, people with CKD and ESKD could be at risk for experiencing unrecognized fatigue exacerbations that result in hospitalization. Routine assessment of patient-reported outcomes (PROs) in clinical care has potential to enhance quality of care, increase quality of life, and improve clinical outcomes^23,24^ and many have recently highlighted the potential benefits of incorporating patient-reported outcome measures (PROMs) into kidney disease research and clinical care.^25-28^ For example, the international Standardized Outcomes in Nephrology (SONG) project recently identified fatigue as 1 of 4 core outcomes that should be reported in all CKD trials, and there is now an initiative underway to develop a validated measurement tool for fatigue in CKD.^6^ The development of such an outcome measure, combined with broader initiatives to introduce routine PROMs into clinical practice, might enable kidney care providers to identify and address fatigue prior to the onset of decline and hospitalization.

As the precipitating factors that led to hospitalizations for fatigue in this study are unknown, it is unclear what interventions might help to prevent them. The use of a nonspecific fatigue diagnostic code as the most responsible diagnosis suggests these hospitalizations likely could not be attributed to a specific underlying diagnosis (eg, anemia, infection). Recent changes in medications or dialysis prescriptions, or missed dialysis sessions, are possible instigators of worsening fatigue that could require imminent care. However, these hospitalizations could also reflect exacerbations of the chronic fatigue often seen in CKD, which is linked to a complex array of factors (eg, inflammation, malnutrition, uremia) that are difficult to diagnose.^29^ The complex etiology of CKD fatigue means there are few evidence-based options for addressing it, beyond erythropoietin stimulating agents, which can reduce fatigue in individuals with low hemoglobin levels.^30^ Exercise training has also been shown to increase vitality levels in both CKD and ESKD,^31,32^ but there are several barriers to its widespread implementation and uptake in this population.^33,34^ Literature from other chronic disease populations^35-37^ suggests that other unexplored self-management based approaches, such as cognitive-behavioral therapy (CBT) and energy management education, could also be beneficial. These approaches teach coping skills, such as emotional management and activity pacing, that promote everyday fatigue self-management. The impact of fatigue self-management programs on hospitalizations has never been investigated, but general disease self-management programs have been shown to reduce hospital admissions.^38,39^ Clinical trials are currently underway to investigate the effects of CBT and energy management education in the ESKD patient population.^40,41^

Hospitalizations for fatigue could also be indicative of a broader frailty syndrome. Frailty is characterized by a combination of symptoms, including fatigue, weakness, and reduced mobility,^42^ that make it more challenging to live independently and increase the risk of hospitalization. There is extensive literature to show that frailty can be addressed via geriatric rehabilitation among older people who are medically stable, non-palliative, and have functional limitations preventing them from living independently at home.^43^ Additional options to support frail individuals with CKD or ESKD in living at home successfully include in-home assistance with activities of daily living, prescription of mobility devices, or home environmental modifications. Further exploration of details regarding the causes, interventions, and outcomes of people with CKD who are hospitalized with fatigue would provide more insight into current practice approaches and opportunities to improve outcomes, particularly as more than 10% of patients hospitalized for fatigue were subsequently hospitalized for fatigue again in the follow-up period.

Our study has several limitations. First, we used a combination of administrative data-derived diagnostic codes that have not been validated to identify fatigue-related hospitalizations; thus, there is a potential for misclassification and underestimation of fatigue-related hospitalization outcomes. However, we considered only the most responsible diagnosis in defining the outcome, and limited the ICD codes to those that were clearly describing fatigue, ensuring face validity, to reduce the potential for misclassification of outcomes. Validation of a diagnostic algorithm for fatigue is an important future research endeavor. Second, our cohort was limited to adults who had at least 1 outpatient serum creatinine measurement or were receiving dialysis, and therefore would not include individuals who did not access the health care system to have a creatinine measurement. Finally, there is the potential for residual confounding, due to the limitations in our dataset and our inability to control for all possible variables (eg, depression) that could confound the association between eGFR and risk of hospitalization for fatigue.

## Conclusions

In this population-based study, we found that reduced eGFR was associated with an increased incidence of fatigue-related hospitalizations across all age groups, and that the difference between patients with CKD and those without was most pronounced among younger adults with more advanced CKD. Further research into methods to address fatigue and frailty in the CKD and ESKD populations is warranted.

## Supplemental Material

sj-pdf-1-cjk-10.1177_20543581211001224 – Supplemental material for The Association Between Estimated Glomerular Filtration Rate and Hospitalization for Fatigue: A Population-Based Cohort Study Click here for additional data file. Supplemental material, sj-pdf-1-cjk-10.1177_20543581211001224 for The Association Between Estimated Glomerular Filtration Rate and Hospitalization for Fatigue: A Population-Based Cohort Study by Janine F. Farragher, Jianguo Zhang, Tyrone G. Harrison, Pietro Ravani, Meghan J. Elliott and Brenda Hemmelgarn in Canadian Journal of Kidney Health and Disease
