# Supplementary material for: The Association Between Estimated Glomerular Filtration Rate and Hospitalization for Fatigue: A Population-Based Cohort Study
Source: Can J Kidney Health Dis. 2021 Mar 16;8:20543581211001224. doi: 10.1177/20543581211001224 (PMC7970172; doi:10.1177/20543581211001224)
Supplement: sj-pdf-1-cjk-10.1177_20543581211001224 – Supplemental material for The Association Between Estimated Glomerular Filtration Rate and Hospitalization for Fatigue: A Population-Based Cohort Study [file sj-pdf-1-cjk-10.1177_20543581211001224.pdf]

**Supplement 1: Incidence rates and rate ratios for fatigue-related hospitalizations, according to age and kidney function**

| Age (years) | eGFR | n | Follow-up person-years | No. hospitalizations with fatigue as most responsible diagnosis | Crude IR per 1000 patient-years (95% CI) | No. hospitalizations with fatigue as secondary diagnosis | Crude IR per 1000 patient-years (95% CI) |
|---|---|---|---|---|---|---|---|
| Age [18,65) | CKD 1&2: eGFR>=60 | 2377132 | 12169423 | 944 | 0.08 (0.07 ,0.08) | 7069 | 0.58 (0.57, 0.59) |
| | CKD 3a: 45<=eGFR<60 | 26266 | 152105 | 66 | 0.43 (0.34 ,0.55) | 271 | 1.78 (1.58, 2.01) |
| | CKD 3b: 30<=eGFR<45 | 5262 | 29813 | 38 | 1.27 (0.93 ,1.75) | 118 | 3.96 (3.30, 4.74) |
| | CKD 4: 15<=eGFR<30 | 2042 | 10321 | 22 | 2.13 (1.40 ,3.24) | 65 | 6.30 (4.94, 8.03) |
| | CKD 5: eGFR<15 (no dialysis) | 719 | 2969 | 5 | 1.68 (0.70 ,4.05) | 33 | 11.12 (7.90, 15.64) |
| | Dialysis | 923 | 3221 | 20 | 6.21 (4.01 ,9.63) | 53 | 16.46 (12.57, 21.54) |
| Age [65,75) | CKD 1&2: eGFR>=60 | 191924 | 1164894 | 856 | 0.73 (0.69 ,0.79) | 3131 | 2.69 (2.60, 2.78) |
| | CKD 3a: 45<=eGFR<60 | 27640 | 169184 | 233 | 1.38 (1.21 ,1.57) | 794 | 4.69 (4.38, 5.03) |
| | CKD 3b: 30<=eGFR<45 | 7229 | 41648 | 109 | 2.62 (2.17 ,3.16) | 348 | 8.36 (7.52, 9.28) |
| | CKD 4: 15<=eGFR<30 | 1773 | 8894 | 46 | 5.17 (3.87 ,6.91) | 148 | 16.64 (14.17, 19.55) |
| | CKD 5: eGFR<15 (no dialysis) | 335 | 1390 | 7 | 5.04 (2.40 ,10.56) | 22 | 15.83 (10.42, 24.04) |
| | Dialysis | 410 | 1343 | 16 | 11.91 (7.30 ,19.44) | 40 | 29.78 (21.84, 40.60) |
| Age>=75 | CKD 1&2: eGFR>=60 | 103320 | 564733 | 1386 | 2.45 (2.33 ,2.59) | 4925 | 8.72 (8.48, 8.97) |
| | CKD 3a: 45<=eGFR<60 | 46644 | 245437 | 849 | 3.46 (3.23 ,3.70) | 2920 | 11.90 (11.47, 12.34) |
| | CKD 3b: 30<=eGFR<45 | 23632 | 110154 | 586 | 5.32 (4.91 ,5.77) | 1925 | 17.48 (16.71, 18.27) |
| | CKD 4: 15<=eGFR<30 | 6764 | 24873 | 199 | 8.00 (6.96 ,9.19) | 625 | 25.13 (23.23, 27.18) |
| | CKD 5: eGFR<15 (no dialysis) | 758 | 2036 | 21 | 10.32 (6.73 ,15.82) | 59 | 28.98 (22.46, 37.41) |
| | Dialysis | 497 | 1476 | 19 | 12.87 (8.21 ,20.18) | 41 | 27.78 (20.45, 37.73) |
